# Parenteral Nutrition in Liver Resection

**DOI:** 10.1155/2012/508103

**Published:** 2012-03-29

**Authors:** Carlo Chiarla, Ivo Giovannini, Felice Giuliante, Francesco Ardito, Maria Vellone, Agostino Maria De Rose, Gennaro Nuzzo

**Affiliations:** ^1^Department of Surgical Sciences, CNR-IASI Center for the Pathophysiology of Shock, Catholic University of the Sacred Heart, Largo Agostino Gemelli 8, 00168 Rome, Italy; ^2^Hepatobiliary Surgery Unit, Department of Surgical Sciences, Catholic University of the Sacred Heart School of Medicine, 00168 Rome, Italy

## Abstract

Albeit a very large number of experiments have assessed the impact of various substrates on liver regeneration after partial hepatectomy, a limited number of clinical studies have evaluated artificial nutrition in liver resection patients. This is a peculiar topic because many patients do not need artificial nutrition, while several patients need it because of malnutrition and/or prolonged inability to feeding caused by complications. The optimal nutritional regimen to support liver regeneration, within other postoperative problems or complications, is not yet exactly defined. This short review addresses relevant aspects and potential developments in the issue of postoperative parenteral nutrition after liver resection.

## 1. Introduction

Although there has been a plethora of animal studies on factors and nutritional substrates supporting or inhibiting liver regeneration after liver resection (LR), a limited number of human studies has addressed parenteral or enteral nutrition in LR. This is a peculiar topic. Indeed, most surgeons do not give particular value to substrate infusion after LR, because most patients can resume an oral diet within the 3rd or the 4th postoperative day, so that starvation does not become an issue. Therefore, it is difficult to find evidence-based information or guidelines on this topic.

On the other hand, major LR, compared to other abdominal operations, is characterized by a specific need for substrate for liver regeneration, and in extreme cases also by competition for substrate to simultaneously support acute phase response, liver regeneration, and host defence if postoperative sepsis occurs [1]. Nutritional support may become an issue not only when the resumption of feeding is prevented by complications and prolonged illness, but also soon postoperatively in malnourished patients with a high risk of complications.

## 2. Randomized Studies

Among the clinical studies which have addressed artificial nutrition in LR in previous decades, there are also randomized protocols comparing parenteral to enteral nutrition after LR, or even preoperatively [2–7]. The results are relevant, and, in particular, one of them clearly shows that artificial nutrition improves the outcome of hepatectomy in cirrhotic patients [2]. However again, these studies are not sufficient to establish realistic indications. Firstly, preoperative artificial nutrition does not comply with the current practice of patient management and hospitalization. Secondly, as already mentioned, after surgery most patients rapidly restart oral feeding without a clear need for artificial nutrition. Thirdly, when artificial nutrition is indicated because of prolonged inability to feeding, there are often various combinations of malnutrition, acute liver dysfunction, and sepsis whose complex management largely overcomes that of routinely recovering LR patients. Up until now, there are no guidelines specifically considering the need to support liver regeneration within other problems and complications, and nutritional prescriptions are still empirically tailored. Although our experience is mostly based on parenteral nutrition, even an evidence-based choice of enteral or parenteral nutrition remains an unsolved issue. The reasonable option is to take advantage of both, when artificial nutrition is indicated, as the former maintains bowel integrity and gut wall barrier function, while the latter allows the obtainment of full-regimen support and fluid-electrolyte balance, which may not always be attainable by enteral nutrition alone.

## 3. Patients with Malnutrition

Important malnutrition [8] may be a reason to delay major surgery for a few weeks if a preoperative improvement in nutritional state is achievable. For instance, malnutrition is sometimes associated with severe obstructive jaundice in patients with hilar cholangiocarcinoma (Klatskin tumor). In these cases the postponement of major hepatectomy may simultaneously be dictated by the need for biliary drainage to treat jaundice, for portal embolization to hypertrophy future remnant liver, and for the improvement of nutritional state [9]. Furthermore, given the high risk of complications and prolonged illness, full-regimen parenteral nutrition is started soon postoperatively. Similar considerations apply to cirrhotic patients after major hepatectomy, in whom liver regeneration and recovery may be more dependent on appropriate nutritional support [10].

## 4. Choice of Substrates

We perform at present about 150 LR per year, and it has long been our standard to administer to all patients from the 1st p.o. day a dose of at least 2.0–2.5 g/kg/day dextrose and 0.8–1.0 g/kg/day amino acids (35 to 50% branched chain amino acids) [9, 11]. This is to moderate the catabolic drive (glycogenolysis, and proteolysis for gluconeogenesis) and to provide some support for liver regeneration, as glucose alone, unless associated with other substrates, does not seem to support hepatic regeneration [12–14]. Intravenous infusions are withdrawn with the resumption of oral feeding, in general within the 3rd or 4th postoperative day.

If a complication prevents feeding, the regimen is augmented to full-dose parenteral nutrition providing 30 to 35 Kcal/kg/day (about 50% dextrose and 50% fat, preferentially using a mixed fat emulsion with 50% medium chain and 50% long chain triglycerides) and about 1.5 g/kg/day amino acids (35 to 50% branched chain amino acids) [9, 11]. A similarly robust support is started soon postoperatively in patients with malnutrition and/or an expected greater risk of complications. This regimen is similar to independently developed regimens adopted for LR in liver cirrhosis, in other patients after major LR and in living donors for liver transplantation [10, 15–17]. Apart from the favourable properties of medium chain triglycerides [10, 18], we simply consider their use as a means of distributing the calorie load over three different substrates (dextrose, long chain, and medium chain triglycerides) therefore avoiding problems associated with high doses of single substrates (difficult glycemic control, excessive load of linoleic acid, etc.). The supply of high doses of branched chain amino acids (BCAAs), within the total amino acid dose, is to take advantage of their moderate anticatabolic and proanabolic effect, and of other favourable properties [10, 19, 20]; there is also long-lasting consensus on their effects in experimental studies on liver regeneration [13, 14, 21]. Of note, BCAA or BCAA-enriched amino acid solutions are not particularly expensive in Italy. 

 Appropriate amounts of electrolytes, vitamins, and trace elements are obviously needed. Within micronutrients, particular attention is given to phosphate supply. It is unclear whether early severe hypophosphatemia after LR depends on increased metabolic consumption, urinary loss, or other mechanisms [22–24]. Anyhow it seems to predict a higher risk of complications, and there is some evidence that phosphate replacement is associated with better outcome, also in living donors for liver transplantation [22, 25]; this needs to be confirmed in further studies.

## 5. Complications

The most common complications after LR are transient liver insufficiency and sepsis. Transient liver insufficiency in itself might not be a reason to modify nutritional support. However, the presence of encephalopathy may involve the reduction or interruption of fat infusion, which can worsen this symptom [18, 26]. A relatively high total amino acid dose (1.0–1.5 g/kg/day) in the presence of liver insufficiency should not be of concern if this include a large percentage of BCAAs. BCAAs do not represent additional burden for the liver because they are primarily metabolized in other tissues; thereafter, the liver advantageously uses their metabolites [19, 20]. High BCAA doses maintain a high plasma BCAA/aromatic amino acid ratio and better control this component of hepatic encephalopathy, maintain some anticatabolic and proanabolic drive, improve energy metabolism, and have other properties which can also include a contribution to reducing ammonia levels [27]. Sometimes in severe encephalopathy there is a need to infuse only BCAAs, interrupting the infusion of other amino acids [28]. Simultaneous dextrose infusion may contribute to increase the BCAA/aromatic amino acid ratio by reducing the endogenous aromatic amino acid load from protein catabolism, while cleaning of the bowel and/or manipulation of its microbial flora reduce endogenous ammonia production.

Unfortunately these supportive measures cannot be of major help when very severe hepatic insufficiency occurs from too small residual liver or other major complications affecting its function. Despite occasional reports on major benefits of nutrition [29], one cannot rely on nutrition to postoperatively enhance function of a small residual liver. The issue must be resolved in advance, by preoperative portal embolization to hypertrophy future remnant liver, or by excluding the patient from resection. In our policy future remnant liver should at least be 25–30% in normal adults and 45–50% in cirrhotic patients, with an intermediate percentage in patients with steatosis or steatohepatitis from chemotherapy before LR for metastases, or in patients with cholestatic liver. Hence, realistically parenteral nutrition after LR can only represent a helpful supportive tool when transient liver insufficiency develops in spite of all precautions.

Liver resection predisposes to sepsis by a variety of mechanisms, including the removal of liver cells involved in host defence [9], and there is well-known synergism of sepsis and liver dysfunction in determining a poor outcome [9, 30, 31]. Of course, treatment of sepsis is the main target, however, nutritional support also has a relevant role. A major surgical procedure which is often associated with septic complications is LR for hilar cholangiocarcinoma. When the patients present with severe obstructive jaundice, there is a need for preoperative biliary drainage which predisposes to microbial contamination of the biliary tree, with a greater risk of sepsis after surgery in a contaminated field. Because surgery often consists of a major or extended hepatectomy, sepsis is often overimposed on transient liver insufficiency [9].

When sepsis develops, the treatment should not diverge much from the common guidelines for sepsis. These include the tailoring of dextrose load according to glucose intolerance, the supply of fat emulsions not containing large amounts of the omega-6 polyunsaturated linoleic acid to avoid excessive amplification of the inflammatory response, and a daily dose of 1.5–2.0 g/kg amino acids (35 to 50% BCAAs). We believe that the use of high-dose insulin to allow large dextrose loads is unsafe in normal surgical wards, because of the risk of hypoglycemic accidents. Reduction of the linoleic acid dose is permitted by using mixed emulsions with 50% long chain and 50% medium chain triglycerides, or olive oil-based emulsions, while additional fish oil emulsions may provide an integration of omega-3 polyunsaturated fatty acids. There are also newly available mixed emulsions containing all these components together (long chain, medium chain triglycerides, and fish oil, with or without olive oil) although published experience on their use in LR is lacking. The total calorie dose (dextrose + fat) should be about 30 to 35 kcal/kg/day. With regard to the combined use of enteral nutrition (whenever feasible) in sepsis, this seems to enhance gut wall barrier function and moderate inflammatory response, while in severe liver insufficiency and encephalopathy enteral nutrition might not be desirable. At any rate, the important concept to emphasize is that, once infection appears, the clinical course could rapidly go downhill. Therefore, parenteral nutrition should be given to patients at risk immediately after LR. In addition, it should be continued even when the patients have resumed oral feeding, which may not be sufficient to cover metabolic demands in the initial period. The patients at greater risk are those with liver cirrhosis, major hepatectomy, and concomitant biliary tract infection or bacterial colonization.

## 6. Biochemical Measurements

**Figure 1:**
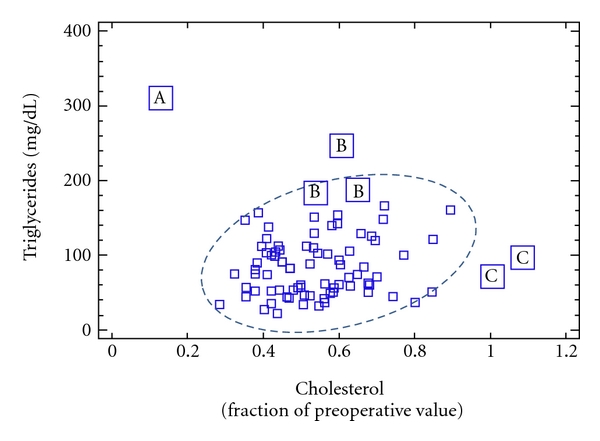
Patients on parenteral nutrition after LR. Within the main group of measurements (encircled), there is general reduction in plasma cholesterol compared to preoperative value, with low-normal triglycerides. A: measurement with the lowest cholesterol and highest triglyceride levels, without fat infusion, signalling the transition of a septic patient to preterminal illness and shock. B: measurements with less severe hypertriglyceridemia signalling transient but reversible worsening of septic illness in two patients undergoing fat infusion. C: measurements with increased cholesterol associated with development of cholestasis in another patient.

There is a well-known series of biochemical measurements which are commonly performed after LR, or in complicated cases undergoing parenteral nutrition, and do not deserve particular notes. Conversely the dosage of plasma cholesterol, triglycerides, cholinesterase, and blood urea nitrogen deserve some comment. Transient hypocholesterolemia is a normal consequence of surgery, and after LR is mainly related to extent of liver resection, severity of acute-phase response, hemodilution from blood loss, and liver dysfunction, if present. Normally hypocholesterolemia progressively recovers. Persistent, or persistently severe hypocholesterolemia after LR has a different relevance, because it is related to sepsis or to a synergic combination of sepsis and liver insufficiency and very poor prognosis [32, 33]. Of note, cholestasis, if present, moderates the degree of hypocholesterolemia. These concepts implicate that persistent hypocholesterolemia after LR has very little to do with nutritional state, or with the infusion of fat. Fat emulsions contain only an irrelevant amount of cholesterol. Conversely, serum triglyceride levels usefully reflect the plasma clearance of the infused fat. In sepsis the observation of normal or low triglycerides does not have particular relevance, while hypertriglyceridemia may reflect both the worsening of the septic state and/or excessive fat load compared to clearance [33, 34]. Indeed, although in sepsis fat is a preferential substrate, in extreme septic illness impaired fat clearance may develop, therefore, determining moderate-to-severe hypertriglyceridemia (Figure 1). The dosage of plasma butyrylcholinesterase (CHE) is another useful adjunct because decreasing CHE, like decreasing cholesterol, may reflect both severity of acute phase response (sepsis) and severity of liver dysfunction [35], with a risk of poor outcome. The practical implication is the need for the aggressive resolution of a septic focus, if present. Of note, the transfusion of fresh frozen plasma or blood can increase CHE independently of the underlying condition. As in the case of cholesterol, the anecdotal relationship between CHE and nutritional state is lost in critical illness although CHE seems to maintain some direct relationship with the amino acid dose [35]. Increased blood urea nitrogen with normal creatinine may commonly reflect protein hypercatabolism (especially in sepsis), bleeding in the gastrointestinal tract, and sometimes renal dysfunction from hypovolemia. It may also reflect increased exogenous amino acid dose; however, it is often gratifying to observe a decrease in blood urea nitrogen, for any given creatinine and amino acid dose, related to increasing doses of dextrose and fat, therefore reflecting a better use of amino acids for protein synthesis.

## 7. New and Related Interventions

Among other aspects, recent information is addressing the benefits of perioperative dextrose and insulin administration in LR (starting already preoperatively) to maintain hepatic glycogen stores; this seems to be associated with better recovery of liver function and also protection from ischemia/reperfusion injury [29, 36]. With regard to the newly developed mixed fat emulsions with long chain, medium chain triglycerides and fish oil, with or without olive oil, it is very likely that LR patients can benefit from each of these fat components while avoiding potential harm from high doses of single fat substrates. As already mentioned, evidence-based information is lacking, and evaluation of these mixed emulsions in the clinical setting has greater priority over ordinary comparisons between the previously available and simpler emulsions. Finally, with regard to BCAAs, other interesting aspects which deserve mention, although beyond the topic of this short review, are the reported benefits of preoperative oral BCAA supplementation on the early response to LR [37], and the better long term outcome associated with protracted oral supplementation with BCAAs and carbohydrates, after LR for cancer in cirrhotic patients [38].

In conclusion, parenteral nutrition in LR patients still remains a poorly defined field. Firstly because most LR patients only need some short-term and light parenteral support, while a formal parenteral nutrition regimen is required in peculiar or complicated cases. Secondly because, despite a plenitude of animal experiments, there is no evidence-based data for the optimal combination of substrates to support liver regeneration and liver function in the clinical setting. This differs from the experimental setting. Indeed, the ability of the liver to regenerate after resection in humans depends on many factors such as the remnant liver size, the quality of the liver remnant, the damage to the liver remnant during and after surgery, concomitant sepsis, and bile duct obstruction or damage. Hence, it is quite difficult to confirm animal experimental findings in patients unless unified groups of patients are included in the studies. Therefore, guidelines remain relatively empirical, and the field remains open to future and more specific investigations.
